# The Development of a Triaxial Cutting Force Sensor Based on a MEMS Strain Gauge

**DOI:** 10.3390/mi9010030

**Published:** 2018-01-15

**Authors:** You Zhao, Yulong Zhao, Xiaohui Ge

**Affiliations:** State Key Laboratory for Manufacturing Systems Engineering, Xi’an Jiaotong University, No. 28, Xianning West Road, Xi’an 710049, China; zhaoyou628@xjtu.edu.cn (Y.Z.); xjgxh1994@stu.xjtu.edu.cn (X.G.)

**Keywords:** cutting force sensor, sensitivity, micro-electro-mechanical system (MEMS) strain gauge, cross-interference, decoupling matrix

## Abstract

Cutting force measurement is a quintessential task for status monitoring during machining. In the past, a number of cutting force sensors have been developed, each featuring a different set of performance advantages. In a pursuit to improve the measuring sensitivity and reduce the cross-interference error, in this paper we propose a triaxial cutting force sensor based on a commercial micro-electro-mechanical system (MEMS) strain gauge. An elastic-sensitive element comprised of two mutual-perpendicular octagonal rings is designed for triaxial cutting force measurement, and a decoupling matrix is derived from static calibration to reduce cross-interference. It can be concluded from static calibration that the sensor’s sensitivity is 0.32 mV/N, 0.32 mV/N, and 0.05 mV/N in triaxial directions, and the proposed decoupling matrix is able to reduce cross-interference error to 0.14%, 0.25%, and 4.42%. Dynamic cutting force measurement shows that the cutting force sensor can reflect the variation of cutting status very well, it is qualified to measure triaxial cutting forces in practical applications.

## 1. Introduction

Cutting force is closely related to machining accuracy, cutting vibration, heat generation, and tool wear during metal machining [1,2], it reflects every slight change in machining status, and it is a significant factor that influences surface roughness, energy consumption, tool life, etc. Thus, measuring cutting force is an effective method for machining condition monitoring in intelligent manufacturing [3] and can provide data support for cutting fault prediction, tool life research, and cutting parameter optimization [4,5].

Cutting force measurement can be generally divided into two parts: indirect measurement and direct measurement. Indirect measurement is mainly realized by tool vibration detection [6,7], motor current measurement [8,9], optical fiber displacement monitoring [10,11], etc. Indirect measurement method is simple for use but is poor in accuracy. In direct measurement, specially designed cutting force sensors are needed. These sensors are based on different working principles such as the resistance strain effect, the piezoelectricity effect, and the capacitance/inductance effect. Among them, strain gauge and piezoelectric cutting force sensors are widely used due to higher accuracy and good reliability [12]. However, owing to the intrinsic contradiction between sensitivity and rigidity, the strain gauge cutting force sensor’s sensitivity is usually sacrificed in order to achieve high rigidity [13]. For example, Yaldiz et al. has reported a strain gauge cutting force sensor whose sensitivity is 0.082 mV/N, 0.122 mV/N, and 0.119 mV/N in triaxial directions [14]. Panzera et al. developed a cutting force sensor with a sensitivity of 1.342 mV/N, 0.684 mV/N, and 0.514 mV/N after 1000× amplification [15]. In addition, octagonal ring type cutting force sensors have become hot research topics. For instance, Yuan et al. developed an octagonal ring structure triaxial cutting force sensor with sensitivities of 215.5 με/kgf, 237.8 με/kgf, and 326 με/kgf [16], and Shan Zhongchen reported an octagonal ring based cutting force sensor with sensitivities of 1.506 με/kgf, 1.121 με/kgf, and 5.711 με/kgf in theoretical calculation [17].

Piezoelectric cutting force sensors are famous for their high sensitivity and high rigidity. Some piezoelectric sensors, such as the 9129A cutting force sensor from KISTLER Company (Winterthur, Switzerland) [18] and the YDC-III89B cutting force sensor produced by Dalian University of Technology (Dalian, China) [19] are already commercial available. However, piezoelectric cutting force sensors are not suitable for measuring stable or static cutting force due to electric charge leakage [20], so high-impedance electric charge amplifiers are needed, but they are expensive.

Cross-interference error is a key factor in judging cutting force measurement accuracy, which is defined as the ratio between a sensor’s undesirable output caused by the force of another direction and the output caused by its measuring direction’s force in full scale, as described in Equation (1). Researchers have substantially contributed to inhibiting cross-interference error; for example, the authors of [14] introduced a strain gauge cutting force sensor with a cross-interference error in the range of 0.54–9.25%, and the KISTLER piezoelectric cutting force sensor’s (type 9219A) cross-interference error is less than 3%. Table 1 illustrates cross-interference indicators of some typical cutting force sensors, where *F_c_*, *F_f_*, and *F_p_* represent the main cutting force, the feeding force, and the thrust force, respectively. Though these sensors have already achieved favorable cross-interference error, it still remains a possibility for improving.

(1) $$C_{error}=\frac{V_{F_{c}\leftarrow F_{f}}}{V_{F_{c}\leftarrow F_{c}}}\times 100\%$$

where *C_error_*, *V_Fc__←__Ff_*, and *V_Fc__←__Fc_* indicates the cross-interference error, the undesirable output of the *F_c_* measuring circuit under the effect of force in the *F_f_* direction, and the full scale output of the *F_c_* measuring circuit under the effect of force in the *F_c_* direction, respectively.

In this paper, a triaxial cutting force sensor using a commercial MEMS strain gauge in order to achieve high sensitivity is introduced, and a decoupling matrix method is proposed to reduce cross-interference error. Details on the sensor design, fabrication, and performance test will be introduced in the following sections.

## 2. Materials and Methods

### 2.1. MEMS Strain Gauge

The contradiction between sensitivity and rigidity in the strain gauge sensor is inherent due to its working principle [13], which can hardly be avoided. Using a strain gauge with a highly sensitive coefficient is a feasible way to obtain a satisfactory sensitivity while keeping high rigidity.

For a section of conductor with a length of *L*, the cross-section area of *S* and resistivity of *ρ*, its resistance value can be calculated as

(2) $$R=\rho \frac{L}{S}\;.$$

Further, it can be expressed as

(3) $$\frac{dR}{R}=\frac{d\rho }{\rho }+\frac{dL}{L}-\frac{dS}{S}=\pi E\varepsilon +\varepsilon -\left(-2\mu \varepsilon \right)=\left[\pi E+\left(1+2\mu \right)\right]\varepsilon =K\varepsilon$$

where *π*, *E*, *ε*, *μ*, and *K* represent the piezoresistive coefficient, elastic modulus, strain value, Poisson’s ratio, and sensitive coefficient of the conductor, respectively. For the metal material, its *πE* value is so small that it can be ignored, and its sensitive coefficient is around 1.5–2; however, for semi-conductive material, its *πE* value is far larger than that of 1 + 2*μ*, and its sensitive coefficient is as large as 70–170. Therefore, a semi-conductive strain gauge based on a MEMS technique was chosen as the transducing element of the cutting force sensor in this study.

**Figure 1:**
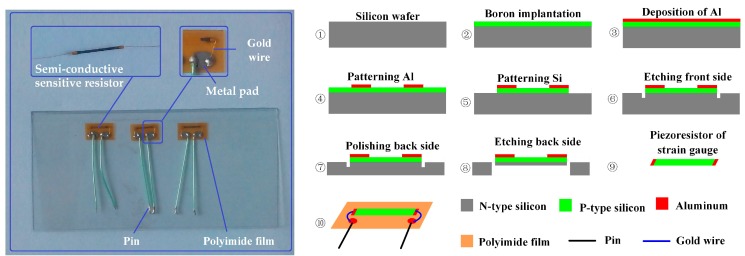
Schematic view of micro-electro-mechanical system (MEMS) strain gauge’s structure and micro-fabrication process.

The MEMS strain gauge (type TP-3-1000) used is a commercial one that was produced by Anhui Tianguang sensor Co., Ltd., Bengbu, China. This kind of MEMS strain gauge has good stability and consistency. It is featured with batch manufacturing and is user-friendly. Figure 1a displays its detailed structure: a semi-conductive sensitive resistor, two gold wires, two metal pads, a polyimide film, and two pins. Detailed parameters of the strain gauge are listed in Table 2 and Table 3. Figure 1b illustrates the fabrication process of the MEMS strain gauge: ① an *n*-type silicon wafer is prepared and cleaned for use; ②boron ions are implanted into the top layer of the *n*-type silicon wafer in order to form a resistance layer (*p*-type); ③ aluminum is sputtered to form a metal electrode layer; ④ the aluminum layer is patterned to form an electrode; ⑤ the resistance layer together with aluminum electrodes are patterned into individual strain gauges; ⑥ the front side of the wafer is etched in order to form a separating groove; ⑦ the wafer thickness is reduced and its back side is polished; ⑧ single resistance pieces are obtained by wet etching the wafer from the back side; ⑨ a single piece of the MEMS strain gauge resistor; ⑩ the semi-conductive sensitive resistor and two pieces of metal pads are glued on the polyimide film, two gold wires are bonded between the resistor and metal pads.

### 2.2. Cutting Force Sensor

**Figure 2:**
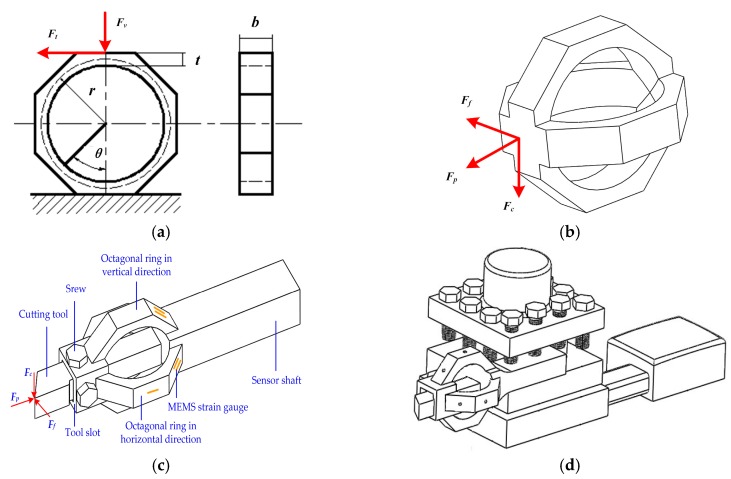
(**a**) Theoretical analysis model of the octagonal ring; (**b**) a schematic view of two mutual-perpendicular octagonal rings; (**c**) the structure and composition of the cutting force sensor; (**d**) the sensor installation on the tool post.

An octagonal ring is a classic elastic-sensitive element (ESE) in sensor design due to its favorable decoupling ability, high rigidity, and good machinability. Thus, octagonal rings are adopted in cutting force sensor design. For an octagonal ring (*t*/*r* ≦ 0.2) under the effect of vertical force *F_v_* and tangential force *F_t_*, as shown in Figure 2a, its surface stress in *θ* = 90° and *θ* = 50° positions can be expressed as follows [14]:

(4) $$\sigma_{90{}^{\circ}\leftarrow F_{v}}=0.7\frac{F_{v}*r}{bt^{2}}$$

(5) $$\sigma_{50{}^{\circ}\leftarrow F_{v}}=0$$

(6) $$\sigma_{90{}^{\circ}\leftarrow F_{t}}=0$$

(7) $$\sigma_{50{}^{\circ}\leftarrow F_{t}}=1.4\frac{F_{t}*r}{bt^{2}}$$

where *t*, *b*, and *r* stand for the thickness, width, and average radius of the ring; *σ*_90°__←_*_Fv_* represents stress in a 90° position caused by *F_v_*, as do *σ*_90°__←_*_Ft_*, *σ*_50°__←_*_Fv_*, and *σ*_50°__←_*_Ft_*.

According to Equations (4)–(7), it is suitable to bond the MEMS strain gauge at 90° and 50° positions on the octagonal ring in order to measure *F_v_* and *F_t_* individually. Considering the requirement of tool installation and high rigidity in practical applications, a structure comprised of two mutually perpendicular octagonal ring (TMPOR) is designed for triaxial cutting force measurement as shown in Figure 2b, where triaxial force components are distributed onto two octagonal rings and each ring is able to measure two of the triaxial components.

The cutting force sensor introduced in this paper is upgraded from our previous work [21,22], Figure 2c depicts the composition of the sensor: the cutting tool is fixed in the tool slot by screws, and the ESE is in the middle of the senor. MEMS strain gauges are bonded on the ESE for measuring triaxial cutting force *F_c_*, *F_f_*, and *F_p_*, respectively. At the end of the sensor is a shaft for fixing it into a tool post, Figure 2d is a schematic view of the sensor’s installation on the tool post.

**Figure 3:**
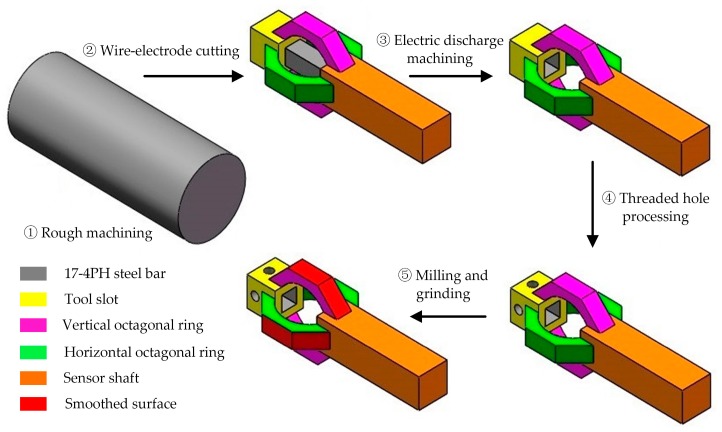
Fabrication process of the cutting force sensor.

17-4PH steel is chosen to fabricate the designed sensor because of its good machinability and mechanical property. The sensor is machined as a monolithic element in order to reduce strain transmission loss and improves the sensor’s response speed. The main processing steps of the sensor are illustrated in Figure 3: ① rough machining and preparing 17-4PH steel with appropriate dimensions for the next step; ② wire-electrode cutting and machining the preliminary shape of the sensor, such as TMPOR and tool shaft; ③ electric discharge machining and removing unexpected columnar structure in the TMPOR; ④ threaded hole processing; ⑤ milling and grinding, removing redundant material, and smoothing strain gauge bonding faces.

### 2.3. MEMS Strain Gauge Bonding

**Figure 4:**
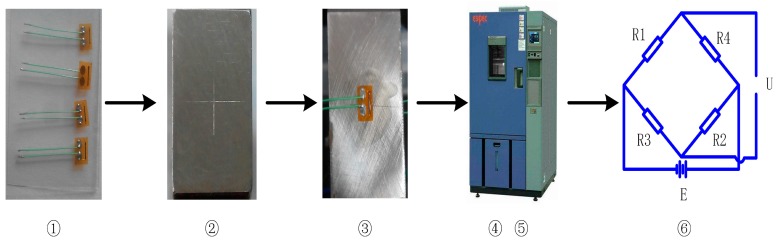
Bonding process of the MEMS strain gauge.

**Figure 5:**
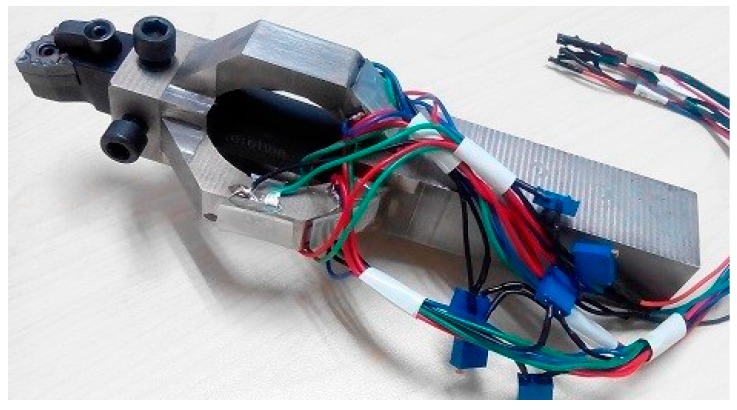
Photograph of the packaged sensor.

The MEMS strain gauge bonding process is a key factor in the sensor’s performance. M-bond 610 glue from Micro-measurement Company is used for MEMS strain gauge attachment. Figure 4 is a schematic view of the bonding process: ① strain gauge selection;—strain gauges with complete structure and close resistance (deviation within ±2 Ω) are selected; ② strain gauge positioning and bonding surface clearance—a cross symbol is marked on the sensor’s surface where strain gauge should be bonded, and the surface is then cleaned by anhydrous ethanol; ③ MEMS strain gauge bonding—a thin layer of M-bond 610 glue is coated on the bonding surface, it is desiccated in an environment of 24 °C with a relative humidity of 50%, and the MEMS strain gauge is then bonded on the pre-coated glue at the marked position, making sure there is no bubble involved; ④ drying and curing—the sensor is placed into an 80 °C environment and dried for 30 min, and it is then put into a 120 °C environment for 2 h of curing; ⑤ post-curing—the sensor is put into a 150 ℃ environment for 1 h in order to achieve a stable bonding and then slowly cooled down to room temperature; ⑥ measurement circuit organization—strain gauges are organized into three groups of Wheatstone bridge for triaxial cutting force measurement. Figure 5 depicts a photograph of the packaged cutting force sensor.

## 3. Results and Discussion

### 3.1. Static Calibration and Result Analysis

**Figure 6:**
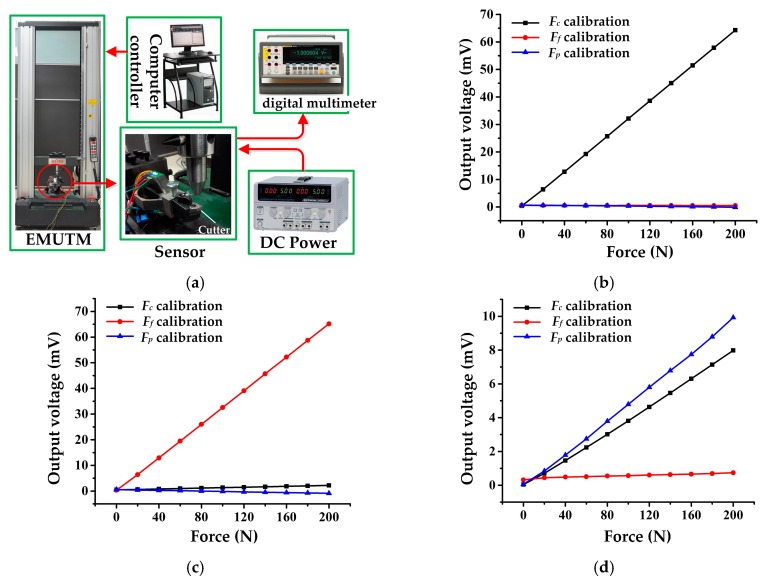
(**a**) Experiment set up for static calibration; (**b**) *F_c_* measurement circuit output in different directions’ calibration; (**c**) *F_f_* measurement circuit output in different directions’ calibration; (**d**) *F_p_* measurement circuit output in different directions’ calibration.

A calibration test was carried out to verify the static performance of the cutting force sensor. An experimental platform was set up, as shown in Figure 6a, including an electro-mechanical universal testing machine (EMUTM, type UTM6104, Suns Company, Shenzhen, China), a computer, a DC power supply (type GPS-3303C, Gwinstek Company, New Taipei City, Taiwan), and three sets of high precision digital multimeter (type FLUKE 8846A, Fluke Corporation, Everett, WA, USA). During the test, the sensor was fixed on the base of EMUTM and powered by 5 V DC. The EMUTM was then controlled by the computer to impose a standard force on the cutter. The standard force increases from 0 to 200 N and then falls from 200 to 0 N in one calibration cycle. Output signals of the sensor were recorded with a high precision digital multimeter. The sensor was calibrated in *F_c_*, *F_f_*, and *F_p_* directions; at least three cycles of test are needed for each direction of calibration, and the experiment data were averaged to diminish random error. Figure 6b–d depict the calibration result for each direction and the main performance indexes of the sensor are listed in Table 4.

Performance indexes of hysteresis, repeatability, linearity, and accuracy are defined as follows:

Hysteresis: The difference between the sensor outputs in the loading and offloading processes is called hysteresis. Hysteresis error can be calculated with Equation (8), where *H* represents hysteresis error; △*y*_max_ is the maximum output deviation between loading and offloading process, and *y*_max_ and *y*_min_ are full scale and zero output signals of the sensor, respectively.

(8) $$H=\pm \frac{\Delta y_{\max}}{2\left(y_{\max}-y_{\min}\right)}\times 100\%$$

Repeatability: The deviation among different groups of sensor outputs under the same input value is called repeatability. Repeatability error can be calculated by Equations (9) and (10). For a certain test cycle, it is assumed that the number of measuring points is m, and the output of each measuring point has been measured n times. Thus, *S_i_* (*i* = 1, 2, …,m) stands for the standard deviation of each measuring point; *y_ij_* is *j-*th (*j* = 1, 2, …,n) measured value of the *i-*th (*i* = 1, 2, …,m) measuring point; *y_i_* is the average value of the measured values for the *i-*th measuring point. *R* stands for the repeatability error; *S*_max_ represents the maximum standard deviation among *S_i_* (*i* = 1, 2, …,m); *y*_max_ and *y*_min_ are full scale and zero output signals of the sensor, respectively.

(9) $$S_{i}=\sqrt{\frac{\sum_{j=1}^{n}{\left(y_{ij}-\bar{y_{i}}\right)}^{2}}{n-1}}$$

(10) $$R=\frac{3S_{\max}}{y_{\max}-y_{\min}}\times 100\%$$

Linearity: Linearity is the maximum deviation between the sensor’s practical output curve and the theoretical curve. In this study, the sensor’s theoretical output curve is obtained by the least-square linear fitting method. Thus, the linearity error can be calculated as Equation (11), where *L* represents the linearity error; △max is the maximum deviation value between the sensor’s practical output curve and its theoretical one.

(11) $$L=\frac{\Delta_{\max}}{y_{\max}-y_{\min}}\times 100\%$$

Accuracy: Accuracy is a comprehensive indicator that reflects the sensor’s static performance; it can be calculated by Equation (12), where *A* represents accuracy error.

(12) $$A=\sqrt{H^{2}+R^{2}+L^{2}}$$

The sensor possesses favorable static performance according to Table 4. Its accuracy is calculated as 3.64%, 1.36%, and 13.82% in triaxial directions. Specifically, its sensitivity is about 27–30 times that of our previous developed metal strain gauge sensor as reported in [21], and it is also higher than that of sensors introduced in the literature [14,15], which proves that using a MEMS strain gauge is a workable method of improving a sensor’s measuring sensitivity. Its sensitivity in the *F_c_* and *F_f_* directions is about five times higher than that of the *F_p_* direction. This is due to the sensor’s ESE structure. According to theoretical analysis of the octagonal ring, the stress value at the 50° position (where the MEMS strain gauges for *F_c_* and *F_f_* measurement were bonded) on the octagonal ring is twice that at the 90° position (where the MEMS strain gauge for the *F_p_* measurement was bonded). As two octagonal rings are involved in the ESE, the stress value at 50° position is four times that at the 90° position, so the sensitivity ratio between the *F_c_* and *F_p_* directions should be four times the theoretical value. Considering the positioning error during strain gauge bonding as well as some unforeseen random errors during the calibration test, this deviation from the theoretical value of the sensitivity ratio between the *F_c_* and *F_p_* directions is reasonable.

In addition, the sensor’s linearity, hysteresis, and repeatability are similar in the *F_c_* and *F_f_* directions, but different with the *F_p_* direction. This is because the sensor’s structure is similar and symmetrical in the *F_c_* and *F_f_* directions, so their performance indexes are almost the same. The difference between the *F_c_* (or *F_f_*) and *F_p_* directions is mainly caused by the fixing method of the cutting tool. As shown in Figure 2c, the cutting tool is fixed in the tool slot by screws, which can hardly avoid relative slippage between the cutting tool and the tool slot when *F_p_* direction force is imposed. This leads to a nonlinear loss of strain and finally causes performance defects.

It is obvious that the cross-interference error in the *F_p_* direction is much higher than those in the *F_c_* and *F_f_* directions, which will seriously affect measuring accuracy. In order to inhibit cross-interference, a decoupling matrix is proposed. Because the linearity error between the sensor’s input and output is as low as 0.46%, 0.48%, and 1.97%, a linear matrix is adopted to describe the input and output relationship of the sensor as follows:

(13) $$\left[\begin{array}{c} V_{F_{c}}\\ V_{F_{f}}\\ V_{F_{p}} \end{array}\right]=\left[\begin{array}{ccc} a_{11} & a_{12} & a_{13}\\ a_{21} & a_{22} & a_{23}\\ a_{31} & a_{32} & a_{33} \end{array}\right]\left[\begin{array}{c} F_{c}\\ F_{f}\\ F_{p} \end{array}\right]+\left[\begin{array}{c} b_{1}\\ b_{2}\\ b_{3} \end{array}\right]$$

where *V_Fc_*, *V_Ff_*, and *V_Fp_* are sensor outputs for *F_c_*, *F_f_*, and *F_p_* measurement circuits; *a_ij_* (*i* = 1, 2, 3, *j* = 1, 2, 3) and *b_k_* (*k* = 1, 2, 3) are constant coefficients. These constant coefficients can be obtained by taking the calibration result into Equation (13), and the constant coefficients are then calculated as listed in Equation (14).

(14) $$\left[\begin{array}{c} V_{F_{c}}\\ V_{F_{f}}\\ V_{F_{p}} \end{array}\right]=\left[\begin{array}{ccc} 0.3207 & 0.298\times 10^{-3} & -0.0037\\ 0.0086 & 0.3259 & -0.0072\\ 0.0400 & 0.0018 & 0.0496 \end{array}\right]\left[\begin{array}{c} F_{c}\\ F_{f}\\ F_{p} \end{array}\right]+\left[\begin{array}{c} 0.4594\\ 0.3431\\ 0.0449 \end{array}\right].$$

According to Equation (14), a triaxial cutting force decoupling equation can be derived as follows:

(15) $$\left[\begin{array}{c} F_{c}\\ F_{f}\\ F_{p} \end{array}\right]=\left[\begin{array}{ccc} 3.0896 & -0.0041 & 0.2299\\ -0.1365 & 3.0661 & 0.4349\\ -2.4867 & -0.1080 & 19.9601 \end{array}\right]\left\{\left[\begin{array}{c} V_{F_{c}}\\ V_{F_{f}}\\ V_{F_{p}} \end{array}\right]-\left[\begin{array}{c} 0.4594\\ 0.3431\\ 0.0449 \end{array}\right]\right\}.$$

In order to verify the feasibility of the decoupling equation in triaxial cutting force measurement, another calibration test was carried out. During the test, a standard static force of 200 N was imposed on the sensor’s tool tip from the *F_c_*, *F_f_*, and *F_p_* directions, respectively. The sensor’s output were recorded, and triaxial cutting force components *F_c_*, *F_f_*, and *F_p_* were calculated from *V_Fc_*, *V_Ff_*, and *V_Fp_* using Equation (15), the calculated cutting force components were compared to the actual standard forces as shown in Table 5.

It can be found from Table 5 that the cross-interference error was effectively reduced using a decoupling matrix, which helps improve the sensor’s measurement accuracy. The general measurement error in each direction are 0.14%, 0.25%, and 4.42%, respectively, when compared to standard input forces, this means that the sensor is capable of accurately measuring triaxial cutting forces, especially in the *F_c_* and *F_f_* directions.

### 3.2. Cutting Force Measurement in the Machining Process

**Figure 7:**
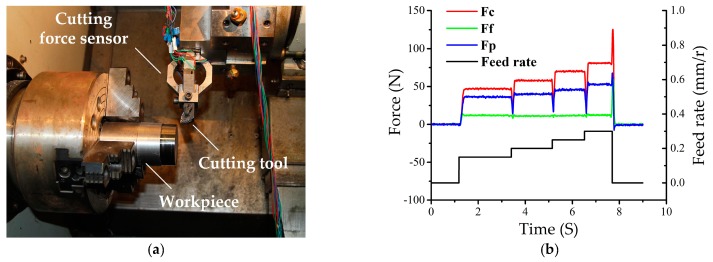
(**a**) Picture of the sensor installed in a CNC lathe; (**b**) cutting force measurement result.

In order to demonstrate the sensor’s practicability for dynamic cutting force measurement, it was installed on a CNC lathe (type FTC-20, Fair Friend Group, Taipei, Taiwan) for the application in the machining process, as illustrated in Figure 7a. Detailed information about the cutting parameters are listed in Table 6. Figure 7b depicts the measured cutting forces versus the change in feed rate. It can be seen that ① cutting force rises promptly with feed rate increments, and they stay in good accordance with each other, which means that the sensor can effectively reflect cutting force variation according to the cutting parameter’s change; ② cutting forces always show an abrupt decrease when feed rate changes, which is because there is a short moment during which cutting declines as the feed rate changes, so cutting force appears to quickly drop; ③ a large peak exists as cutting ends, which is because, when the continuous movement between the cutting tool and the workpiece suddenly stops, there is a sudden increase in cutting force.

## 4. Conclusions

This paper proposes a triaxial cutting force sensor that aims to improve sensitivity and reduce cross-interference error. Static calibration and dynamic cutting force measurement results have demonstrated the feasibility of this proposal. Conclusions can be summarized as follows:

(1) The proposed sensor effectively improves the cutting force sensor’s sensitivity using the MEMS strain gauge. Static calibration result shows that the sensor’s sensitivity is 27–30 times greater than previously developed sensors.
(2) The decoupling matrix is a feasible method of inhibiting cross-interference and helps reduce cross-interference error in the range of 0.14–4.42%, which increases the measurement accuracy of the sensor.
(3) During the cutting force measurement experiment, the measured cutting forces were in good accordance with the change in cutting parameters, which proves that the sensor can reflect cutting status variation very well.

This study has proposed a highly sensitive and low cross-interfering cutting force sensor based on a kind of MEMS strain gauge and a decoupling matrix method. It is simple and effective, as demonstrated in static and dynamic experiments. The developed sensor is competitive due to its concise structure and low cost. Future work will focus on MEMS strain gauge bonding and sensor structure optimization to improve accuracy further. Compared to other piezoelectric cutting force sensors currently in use, our proposed sensor is indeed promising, and continuative study is merited.

## Figures and Tables

**Table 1:** Cross-interference error of some typical cutting force sensors.

Cutting Force	Cross-Interference Error (%)											
	Literature [16]			Literature [17]			Literature [14]			Literature [18]		
	*F_c_*	*F_f_*	*F_p_*	*F_c_*	*F_f_*	*F_p_*	*F_c_*	*F_f_*	*F_p_*	*F_c_*	*F_f_*	*F_p_*
*F_c_*	-	1.29	1.63	-	1.70	2.10	-	3.35	0.54	-	≤3	≤3
*F_f_*	3.64	-	4.00	2.10	-	1.30	1.80	-	0.72	≤3	-	≤3
*F_p_*	3.56	0.50	-	2.00	3.10	-	1.73	9.25	-	≤3	≤3	-

**Table 2:** Physical parameters of the micro-electro-mechanical system (MEMS) strain gauge—Part 1.

Strain Gauge Type	Substrate Size (mm)	Resistor Size (mm)	Resistance (Ω)	Sensitive Coefficient
TP-3-1000	5 × 3	3 × 0.20 × 0.04	1000	150 ± 5%

**Table 3:** Physical parameters of the MEMS strain gauge—Part 2.

Resistance Temperature Coefficient (1/°C)	Sensitivity Temperature Coefficient (1/°C)	Working Temperature (°C)	Working Current (mA)	Strain Limit (με)
<0.40%	<0.30%	<80	5	6000

**Table 4:** Static performance indexes of the sensor.

Cutting Force Component	Static Performance Indexes						
	Sensitivity	Linearity Error	Hysteresis Error	Repeatability Error	Cross-Interference Error		
					*F_c_*	*F_f_*	*F_p_*
*F_c_*	0.32 mV/N	0.46%	0.17%	3.61%	-	2.66%	80.40%
*F_f_*	0.32 mV/N	0.48%	0.35%	1.22%	0.19%	-	4.27%
*F_p_*	0.05 mV/N	1.97%	4.45%	12.93%	1.19%	2.22%	-

**Table 5:** Experiment result for triaxial cutting force decoupling verification.

Standard Force	Measured Forces			Cross-Interference Error			General Error
	*F_c_*	*F_f_*	*F_p_*	*F_c_*	*F_f_*	*F_p_*	
*F_c_* = 200 N	199.73 N	0.875 N	4.750 N	-	0.44%	2.49%	0.14%
*F_f_* = 200 N	1.445 N	199.507 N	6.931 N	0.72%	-	3.63%	0.25%
*F_p_* = 200 N	2.518 N	1.754 N	191.154 N	1.26%	0.88%	-	4.42%

**Table 6:** Experiment parameters of dynamic machining process.

Workpiece Material	Cutting Parameters			
	Workpiece Diameter	Spindle Speed	Depth of Cut	Feed Rate
AISI 1045 steel	61.8 mm	900 r/min	0.1 mm	0.15, 0.20, 0.25, 0.30 mm/r
